# Nodular and diffuse spindle cell infiltration in keloidal scleroderma: a case report

**DOI:** 10.3389/fmed.2023.1291941

**Published:** 2023-12-18

**Authors:** Yiting Li, Yibin Zeng, Zile Chen, Shu Nie, Zhouwei Wu

**Affiliations:** ^1^Department of Dermatology, Shanghai General Hospital, Shanghai Jiao Tong University School of Medicine, Shanghai, China; ^2^Department of Dermatology, Minhang Hospital, Fudan University, Shanghai, China

**Keywords:** keloidal scleroderma, spindle cell infiltration, CD34, nephrogenic systemic fibrosis, case report

## Abstract

Keloidal scleroderma is a variant of scleroderma that presents as firm keloidal nodules or plaques. Due to the similarity in morphology and pathology, it is often distinguished from a hypertrophic scar or keloid. We report a case of keloidal scleroderma with rare nodular and diffuse spindle cell infiltration in histopathology. Recognition of this unusual histopathological feature may help clinicians improve their knowledge and avoid misdiagnosis.

## 1 Introduction

Keloidal scleroderma is a rare variant of scleroderma, characterized by asymptomatic or pruritic keloidal nodules or plaques. Histopathologically, it often shows the features of keloid, scleroderma, or both. In this study, we present a case of keloidal scleroderma with hypercellular spindle cell infiltration in the dermis, which needs to be distinguished from other sclerotic diseases such as nephrogenic systemic fibrosis. Recognizing this unique histopathological feature can help clinicians and pathologists better understand the features of keloidal scleroderma and reduce misdiagnosis.

## 2 Case presentation

**Figure 1:**
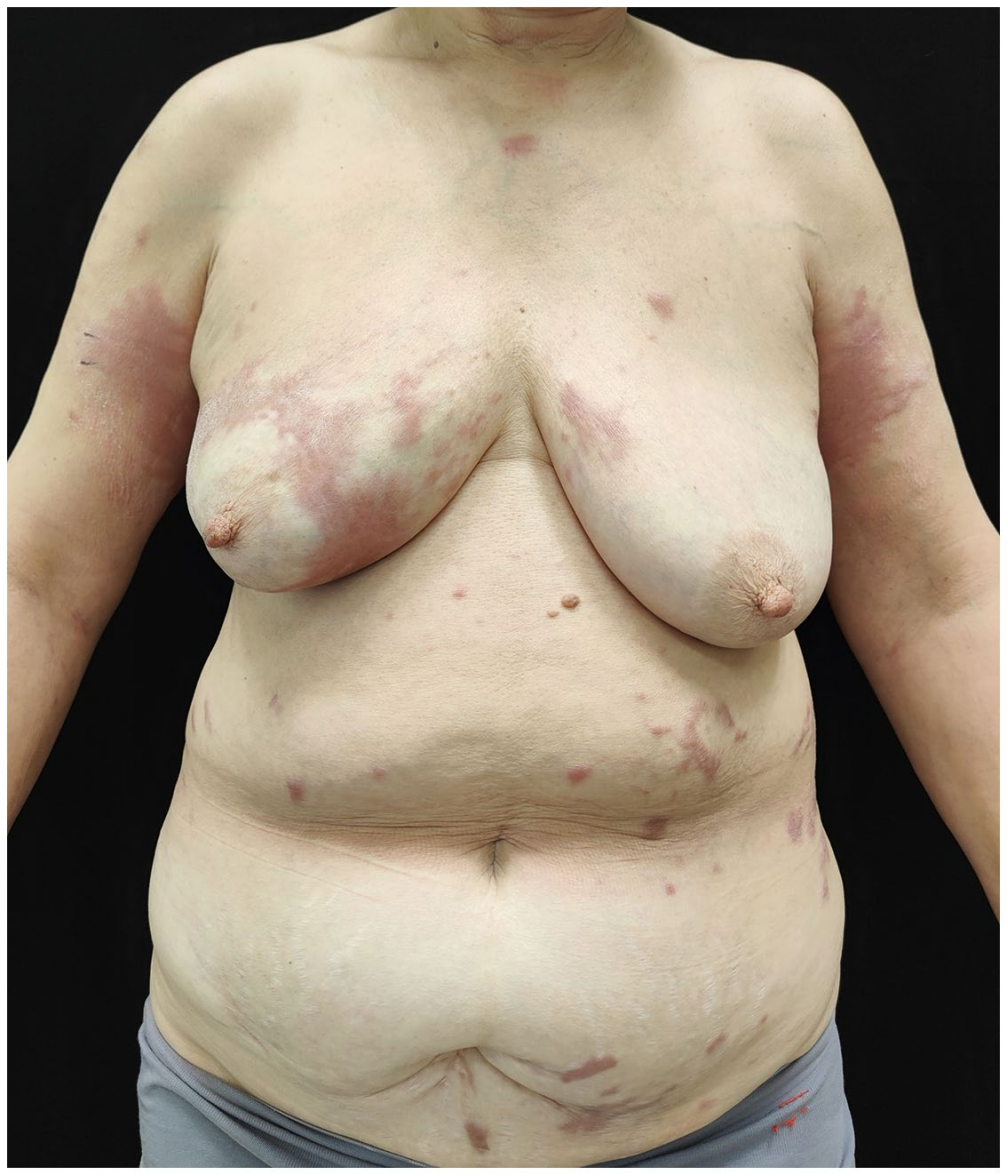
Multiple infiltrated erythematous papules and plaques on her arms and trunk.

**Figure 2:**
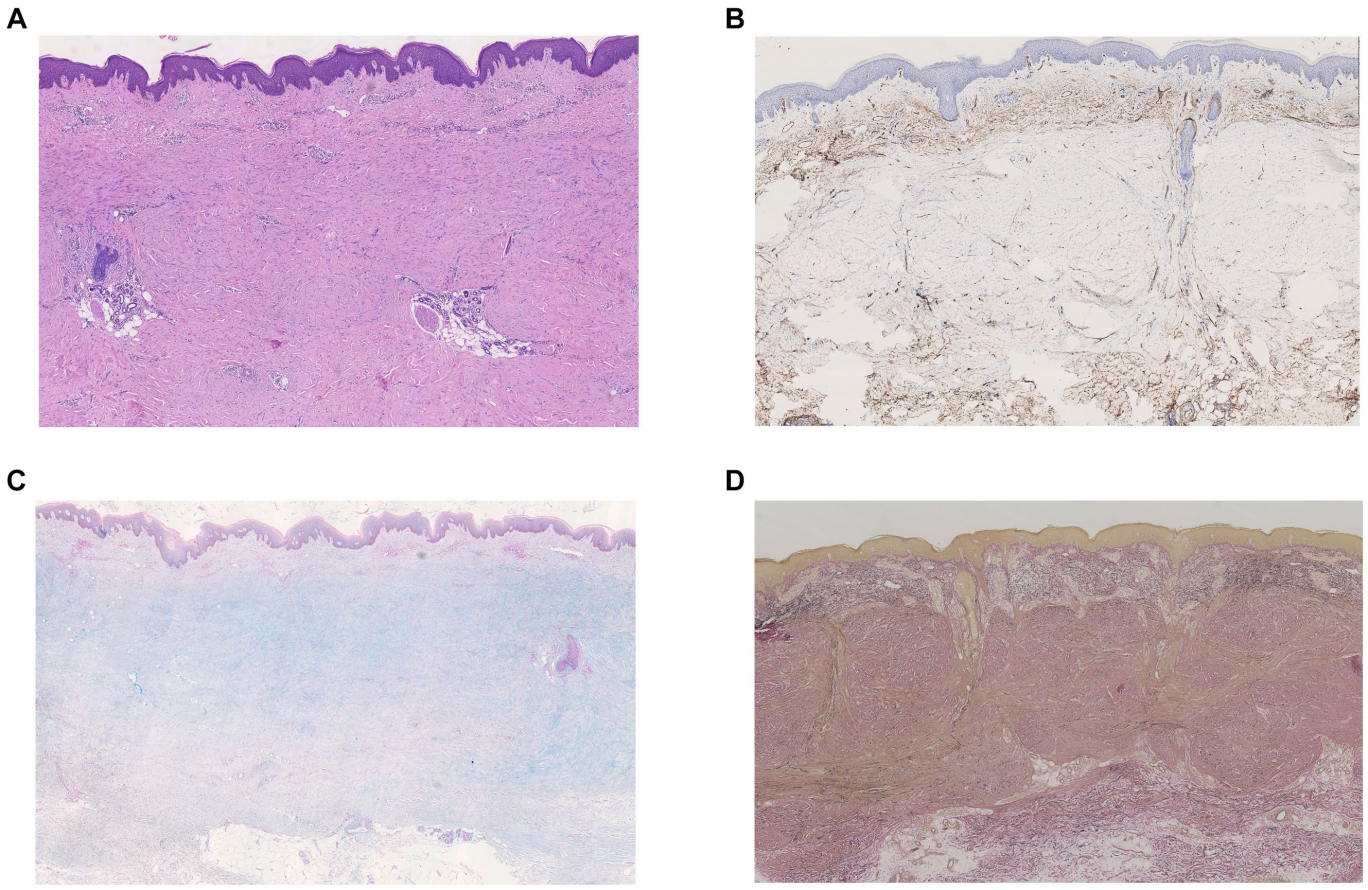
**(A)** Biopsy of the right upper arm and breast showed nodular and diffuse spindle cell infiltration with collagen hyperplasia and perivascular lymphocytic inflammation in the dermis (hematoxylin–eosin, original magnification×40). **(B)** Immunohistochemical staining showed loss of CD34^+^ dermal dendritic cells in dermis (CD34^+^ staining, original magnification × 40). **(C)** Alcian blue staining showed mucin deposition (original magnification×40). **(D)** Verhoeff–Van Gieson staining showed the loss of elastin fibers (original magnification × 40).

A 60-year-old woman presented with a 2-year history of slowly enlarged erythematous rash with itch on the trunk and arms. Her previous medical history was unremarkable, and she denied a history of trauma and exposure to gadolinium-based contrast agents. Physical examination showed multiple, infiltrated erythematous papules and plaques with sclerosis and tenderness involving the arms, anterior chest, and abdomen (Figure 1). Inspection of the patient’s hands revealed red, puffy fingers with abnormal nail fold capillary dilation and hemorrhage. Raynaud’s phenomenon was not found. Laboratory investigations showed that the patient was positive for antinuclear antibody (titer of 1:320) but negative for any other specific autoantibody. Her complete blood counts, liver, renal function, and gammopathy screening were normal. A computed tomography scan of the chest revealed multiple cyst formations and interstitial thickening in both pulmonary. The pulmonary function test was normal. Two biopsy specimens taken from the right upper arm and breast demonstrated nodular and diffuse spindle cell infiltration with collagen hyperplasia and perivascular lymphocytic inflammation in the dermis (Figure 2A). Immunohistochemical staining showed that the spindle cells were negative for CD34 and cytokeratin, and mucin deposition was identified by Alcian blue staining (Figures 2B,C). Verhoeff–Van Gieson staining demonstrated the loss of elastin fibers (Figure 2D). A diagnosis of keloidal scleroderma was made, and potential systemic involvement was suspected. The patient was treated with methotrexate at a dose of 7.5 mg every week.

## 3 Discussion

Keloidal scleroderma is a rare variant of scleroderma, occurring with either progressive systemic scleroderma or localized morphea, which shows a preference for females (1). Multiple factors, including cytokines, cartilage oligomeric matrix proteins, and growth factors such as epidermal growth factors, tenascin, connective tissue growth factor, and transforming growth factor beta, have been reported to play crucial roles in pathogenesis (2). Clinically, it presents as firm nodules, papules, or plaque affecting the trunk or proximal extremities, which may be indistinguishable from a hypertrophic scar or keloid.

The histopathology of keloidal scleroderma shows keloidal or sclerodermatous features. Specifically, the keloidal pattern shows brightly eosinophilic or glassy collagen bundles organized haphazardly in the dermis, while scleroderma shows thick sclerotic collagen bundles arranged parallel to the epidermis with perivascular lymphocytic and plasmacytoid infiltration (3). The differential diagnosis includes hypertrophic scar, keloid, and scleromyxedema. In the present case, hypercellular spindle cell infiltration is very rare, which needs to be differentiated from other sclerotic disorders, particularly nephrogenic systemic fibrosis. Immunohistochemical staining for CD34 helps diagnosis based on the CD34 positivity in nephrogenic systemic fibrosis and the loss of CD34^+^ cells in keloidal scleroderma. In addition, due to the preservation of eccrine sweat glands, this case also needs to be differentiated from dermatomyofibroma. Verhoeff–Van Gieson staining can help distinguish them (4).

Topical corticosteroids, calcipotriene, tacrolimus, imiquimod, systemic methotrexate, steroids, phototherapy, extracorporeal photochemotherapy, and surgical excision are mentioned as treatment choices in the literature, showing unsatisfactory results (5). New therapeutic strategies are currently undergoing investigation. We present this case to help clinicians and pathologists improve their understanding of the unique clinicopathologic features.

## Data availability statement

The original contributions presented in the study are included in the article/supplementary material, further inquiries can be directed to the corresponding author/s.

## Ethics statement

Written informed consent was obtained from the individual(s) for the publication of any potentially identifiable images or data included in this article.Written informed consent was obtained from the participant/patient(s) for the publication of this case report.

## Author contributions

YL: Conceptualization, Data curation, Writing – original draft. YZ: Funding acquisition, Investigation, Writing – review & editing. ZC: Investigation, Writing – review & editing. SN: Investigation, Writing – review & editing. ZW: Conceptualization, Writing – review & editing.
